# Factors Affecting Changes in the Mental Health of North Korean Refugee Youths: A Three-Year Follow-Up Study

**DOI:** 10.3390/ijerph18041696

**Published:** 2021-02-10

**Authors:** Yoanna Seong, Subin Park

**Affiliations:** Department of Research Planning, Mental Health Research Institute, National Center for Mental Health, Seoul 04933, Korea; ynnn0208@korea.kr

**Keywords:** North Korean refugee youths (NKRYs), depression, emotional regulation strategy, expressive suppression, resilience, life satisfaction

## Abstract

This study identified factors affecting changes in depression of 64 North Korean refugee youths (NKRYs) aged 13 to 23 years (40 female) using follow-up data over a three-year period. We collected intrapersonal factors (emotional regulation strategies, resilience, quality of life) and external factors (psychological and practical support, family adaptation, and cohesion) to understand the preventative and risk factors affecting changes in depression. The trend of depression symptoms significantly increased, and the proportion of people classified as depressed (cut-off score = 21) increased steadily from 45.3% to 59.4% in the third year. In addition, we conducted a panel regression analysis, which showed that individual internal factors had a statistically significant effect on changes in depression. Specifically, expressive suppression of emotions was shown to increase depression over time. Resilience and life satisfaction were significant factors reducing depression in this study. On the other hand, external factors were not significantly related to changes over time in depression of NKRYs. Interventions for NKRYs at risk of depression are necessary and should include ways to enhance resilience and life satisfaction, and foster ego strength by recognizing emotions and promoting healthy emotional expression.

## 1. Introduction

According to a report by the Ministry of Unification (September 2020) [1], there are 33,718 North Korean refugees (NKRs) living in South Korea. Among them, a significant proportion, 40% (13,367), are adolescents and young adults aged 10–29. North Korean refugee youths (NKRYs) are likely to have experienced various physical and psychological traumas in North Korea and are known to experience various direct and indirect traumas from the process of defection to South Korea [2,3]. Additionally, one of the latest trends regarding NKRYs is the growing proportion of teenagers born in third countries to parents who are NKRs [4]. In 2020, the proportion of NKRYs born in a third country, including China, was 62.8% [5], and they have been shown to experience difficulties with both language communication and cultural differences [6]. Considering that NKRYs will play a socioeconomic role as members of South Korean society in the future, their stable adaptation is critical.

The cultural adaptation stress experienced by NKRYs in the process of settling is a significant factor that makes adapting to South Korean society difficult [7]. In particular, it is highly likely that they will experience additional stress due to the developmental transition from childhood to adulthood [8,9]. NKRYs are known to experience many psychological symptoms, such as posttraumatic stress disorder, anxiety and depression, delinquency, aggression, and hostile behavior [10,11,12,13,14]. Among them, depression is the most commonly observed psychological problem in NKRYs.

Depression, along with posttraumatic stress disorder, has been used as an indicator of the mental health of these groups, and is commonly experienced by immigrants and refugees [15]. High levels of depression have been reported in NKRYs who perceive their daily stress to be high [11]. Low resilience and low self-esteem are known to be factors that maintain depression [16]. Particularly, resilience is one’s capacity for adapting successfully in spite of adversity or overwhelming circumstances, and its importance as a protective factor for depression was highlighted in a South Australian study with refugee youths [17,18]. Furthermore, low levels of life satisfaction and low expectations of the future were also found to be factors related to depression in NKRYs [19]. Expressive suppression is one of the emotional regulation strategies in which one intentionally suppresses emotionally expressive behavior while experiencing emotional stimulation [20]. It has been shown to exacerbate the effects of early trauma on depressive symptoms [21]. In terms of external factors, low levels of emotional or practical social support contribute to depression in NKRYs [11,19]. On the other hand, several studies have found that an internal locus of control or family support protect against depression in NKRYs and have been reported to be able to help them adapt to living in a new society [8,9,22,23,24]. In addition, participation in social groups (e.g., religious and social organizations) was also found to reduce depression [25].

A two-year follow-up study with 1348 Southeast Asian refugees found that pre-immigration stressors affected the initial mental health of migrants, but over time, the effects of post-settlement stressors, such as financial problem or cultural adaptation were greater [26]. A three-year follow-up study with 151 NKRs in South Korea also showed that experiencing stress after settling in South Korea, such as misunderstanding the language or law, and lack of basic knowledge needed in everyday life, has a stronger impact on depression than the psychological trauma experienced during the defection process [27]. These studies suggest that factors affecting depression of NKRYs may vary over time, and it is therefore necessary to conduct a longitudinal follow-up study taking into account temporal changes. According to a literature review by Lee, Lee, and Park [28], most studies on NKRs used cross-sectional research methods, with very few longitudinal studies to consider changes over time. Furthermore, previous studies have only identified the relationship to each individual factor, but no studies have so far been found to identify relative influences by simultaneously looking at internal and external factors affecting NKRYs’ mental health. The specific purpose of this study is to answer the following questions: First, do the symptoms of depression in North Korean defectors change over time? Second, what are the risk factors or protective factors that affect changes in their symptoms of depression? Thus, the present study seeks to find a longer-term way to intervene in the psychological problems of NKRYs by clarifying the factors affecting changes over time, using three-year follow-up data. In addition, this study clearly identified intrapersonal and external factors, as well as the preventative and risk factors affecting depression.

## 2. Method

### 2.1. Participants

Participants were recruited from two alternative schools for NKRYs in Seoul. Both schools participated voluntarily in the study for the benefit of the mental health screening program. Our research team visited each school and asked for consent to participate in the study after explaining the contents of the questionnaire and the procedures of this research. Furthermore, in the case of participants under the age of 20, the consent of the participant and his/her parents was obtained together. All participants were firstly provided with questionnaires in Korean. However, for participants whose main language is Chinese, questionnaires with Chinese translations were provided, and questionnaires without Chinese versions were directly explained to and answered by Korean and Chinese bilingual speakers. Among all students who attended the two schools, 174 students enrolled in our study in 2017 and 2018 (baseline). A total of 108 completed the following year’s questionnaire (T2), and only 64 were finally included in the study after completing a three-year follow-up questionnaire in 2018–2019 and 2019–2020 (attrition rate: 63.29%), because of changes such as graduation, suspension of study, and relocations.

Sociodemographic information and childhood trauma experience were included in the baseline questionnaire. In addition, depression, emotional regulation strategies, resilience, life satisfaction, psychological and practical support, family adaptation, and cohesion were reported (T1). One year later, follow-up data were collected using an identical questionnaire (T2), and the year after, the second set of follow-up data were accumulated (T3). The study was reviewed and approved by the institutional review board of the National Center for Mental Health (No. 116271-2017-11).

### 2.2. Measurements

#### 2.2.1. Sociodemographic Characteristics

Sociodemographic data were collected including age, gender, period of living in South Korea, country of birth, and residential type (i.e., with the immediate family, other relatives, friends, alone, in a dormitory, or in a facility).

#### 2.2.2. Childhood Trauma Experience

Early trauma experiences were assessed using the Adverse Childhood Experiences (ACE) questionnaire [29]. The ACE questionnaire consists of 17 items that assess whether participants had ever experienced various adverse childhood experiences and dysfunctional family relations, including child abuse (6 items), neglect (4 items), and household dysfunction (7 items) such as domestic violence and mental illness (1 = yes, 0 = no). The total score ranges from 0 to 17, with higher scores indicating more experiences of early trauma. Cronbach’s α for the scale was 0.80.

#### 2.2.3. Depression

Depression symptoms were measured using the Center for Epidemiologic Studies Depression Scale (CES-D), which was developed to measure depressive symptoms in the general population [30]. The Center for Epidemiology Studies-Depression Child Scale (CES-DC) [31] was used for individuals younger than 19 years. Both scales were translated into Korean and validated with the Korean population [32,33], and we utilized the Korean versions in this study. Also, Chinese validated version of CES-D and CES-DC were provided for participants whose main language is Chinese [34,35]. The CES-D and CES-DC are comprised of 20 items: four positive items and sixteen negative items. The scale was rated on four points: 0 = rarely or none of the time (less than one day per week), 1 = occasionally (one or two days per week), 2 = frequently (three–four days per week), and 3 = most or all of the time (five–seven days per week). Higher scores indicate greater depressive symptoms; total scores reflect depression [36]. In the present sample, Cronbach’s α for the scale was 0.85.

#### 2.2.4. Protective and Risk Factors: Intrapersonal Factors

Emotion regulation strategies were assessed using the Emotion Regulation Questionnaire (ERQ) [37], which comprises 10 items measuring two emotion regulation strategies: cognitive reappraisal (6 items) and expressive suppression (4 items). The original version of the ERQ was rated using a seven-point Likert scale (1 = strongly disagree, 7 = strongly agree). In this study, we adopted the validated Korean version of the ERQ, which has been modified into a five-point Likert scale to make it easier to answer [38]. The total scores range from 6 to 30 for cognitive reappraisal and 4 to 20 for expressive suppression, with higher scores indicating greater use of the corresponding emotion regulation strategy. We used each sub-item separately, Cronbach’s α for cognitive reappraisal was 0.76, and for expressive suppression was 0.58 in the present study.

Resilience was assessed using the Brief Resilience Scale [39], which is the self-perceived ability to recover from stress. The six items were scored on a five-point scale (1 = strongly disagree, 5 = strongly agree). The scale consists of three positive and three negative items. The range of total possible scores is from 6 to 30, and a higher score represents higher resilience. In this study, Cronbach’s α for the scale was 0.79.

Life satisfaction was measured by the subjective well-being question from the Gallup World Poll [40], which was originally from the Cantril Self-Anchoring Striving Scale [41]. Participants describe their level of life satisfaction during the past, the present, and the future on a scale of 0 to 10 (0 = worst, 10 = best); we used only the response pertaining to the present.

#### 2.2.5. Protective and Risk Factors: External Factors

Psychological support was assessed by the following question: “How much psychological support do you currently receive from your family, relatives, friends, and others around you?” Practical support was assessed by the following question: “How much practical support do you currently receive from your family, relatives, friends, and others around you?” The responses to both questions were assessed using a 10-point Likert scale (1 = not at all, 10 = receive enough support).

The family adaptability and cohesion evaluation scale III (FACES-III) was developed by Olson, Portner, and Lavee [42] to assess family function for family cohesion and adaptability. In this study, a measure validated in Korean by Lim, Lee, Oh, Kwak, Lee, and Yoon [43] was used. It consists of two sub-scale units of adaptability and cohesion. Family adaptability indicates the degree to which the family system can change in response to current and developmental stresses facing the family system, and family cohesion represents emotional bonds between family members. Each consists of 10 questions on a 5-point Likert scale. The higher the score, the higher the cohesion and adaptability of the family. The Cronbach’s α of family adaptation was 0.91 and family cohesion was 0.9 in the present study.

### 2.3. Statistical Analysis

Descriptive statistics were conducted to describe the sociodemographic characteristics of the data at the baseline time. Repeated measures analysis of variance (ANOVA) was conducted to reveal the annual differences of each variable, using SPSS 20.0 (IBM SPSS Statistics for Windows, version 20.0; IBM: Armonk, NY, USA, 2011). For panel regression analysis, the longitudinal data (wide type) collected over three years were merged first, and the data converted to long type data according to the ID and utilized for analysis. Additionally, a Hausman test confirmed the suitability of the fixed effect model and the random effect model. The Hausman test considers it more appropriate to apply a random effect model if the null hypothesis is rejected, rather than applying a fixed effect model [44]. The Hausman test and panel regression analysis were performed using STATA 14.0 (StataCorp LCC, version 14.0; Texas, USA, 2015.) with a statistical significance level of α = 0.05.

## 3. Results

Table 1 presents the characteristics of the 64 participants. The sample was composed of 40 females (62.5%) and 24 males (37.5%) aged 13 to 23 years (mean = 16.89, SD = 1.64 years). At the time of their first enrollment, the average number of years they had lived in South Korea was 3.06 years (SD = 2.54), from less than one year to 12 years. Meanwhile, the average childhood trauma experience was 1.63 (SD = 2.38), with 42.2% reporting no experience of trauma.

Descriptive statistics and repeated measures ANOVA were performed to identify the annual characteristics of variables; results are presented in Table 2. The trend for depression symptoms increased year by year, and was also statistically significant (F = 3.09, *p* < 0.05). Specifically, the proportion of people who can be classified as having depression (cut-off score 21) increased steadily from 45.3% in the first year, to 53.1% in the second year, and 59.4% in the third year. Satisfaction with life was not statistically significant, but was observed to be decreasing, and practical support was also perceived to be decreasing by participants compared to the first year, indicating that the statistical differences are significant (F = 3.516, *p* < 0.05).

A panel regression analysis was performed to determine how much change in the independent variables over time affects the degree of change in depression, and the results are presented in Table 3. Prior to the panel regression analysis, the Hausman test was performed. As a result, the random effect model was adopted in this study, indicating that the probability of significance was greater than 0.05 (χ^2^ = 14.69, *p* = 0.1). The overall model’s explanatory capacity was 37%, suggesting that the model’s explanatory power was high (R^2^ = 0.37, *p* < 0.01). First, it was shown that suppression of emotional expression affects depression as the period of living in South Korea increases. In other words, if emotional expression suppression increases by one unit over time, depression increases by 0.89 (B = 0.886, *p* < 0.05). On the other hand, resilience and present life satisfaction have been shown have an opposite effect over time, and with each unit increase in resilience and life satisfaction, depression decreased by 0.87 and 0.8 (B = 0.867, *p* < 0.001; B = 0.798, *p* < 0.05). The correlation between the independent variables and depression at each time point is presented in the Supplementary Table S1. Meanwhile, external factors (i.e., emotional and practical support, family adaptability, and cohesion) were not significantly related to changes over time in the depression of NKRYs.

## 4. Discussion

This study aimed to identify the factors affecting changes in depression over time by tracking NKRYs for three years. The findings and implications of this study are presented below.

First, the depressive symptoms of NKRYs increased significantly over the three years. Consistent with our results, a three-year follow-up study conducted by Cho et al. [27] with NKRs also showed that the level of depression increased significantly over three years after settling in South Korea. In addition, the number of participants who could be classified as depressed based on the cut-off score also increased every year, especially in the third year, with a high rate of about 60%. This is higher than that reported in previous cross-sectional studies with depression rates of approximately 30% to 48% [13,19,45], indicating that the psychological vulnerability of NKRYs can be considered high. Several studies have pointed to academic and socio-cultural differences as some of the reasons why NKRYs experience difficulties in adapting [46,47]. Despite the importance of academic achievement due to their developmental age, many NKRYs give up their regular academic courses because of academic maladjustment and the burden of academic achievement, which is likely to be high [46]. In addition, they are reported to have difficulty in forming peer relationships because of acculturation stresses based on different values and forms of expression of opinions, which may consequently lead to deterioration in mental health [48]. After arriving in South Korea, they receive short-term adaptation education and are immediately deployed to South Korean society, and their psychological stress seems to be increasing as they enter a boundlessly competitive system with South Korean teenagers without psychological stability and adaptation. [14]. In particular, given that the average residence period in South Korea is only 3.06 years, special attention is required for the mental health of NKRYs in the early stages of settlement.

Second, panel regression was used to determine factors affecting changes in depression over time, and it was shown that individual internal factors had a statistically significant effect on changes in depression. Specifically, expressive suppression of emotion was shown to increase depression over time. This result is in line with previous studies that mentioned expressive suppression as a risk factor for depression [16,21]. Aldao, Nolen-Hoeksema, and Schweizer [49] suggested that the presence of expressive suppression is likely to be more strongly associated with depression than the absence of cognitive reappraisal. In terms of interpersonal relationships, adolescents seem to choose emotional suppression rather than expression to avoid damaging relationships with peers who show their own symptoms of depression [50].

In addition, resilience was a significant preventative factor for changes in depression in this study. Previous studies on NKRYs focused on resilience as a preventative factor for mental health issues including depression [16,19,24,45,51]. According to McLaughlin, Doane, Costiuc, and Feeny [52], resilience consists of two aspects: heightened psychological vulnerability and adaptation to risk. Based on the above concepts, for NKRYs who are forcibly exposed to various stresses, resilience is likely to be an important internal factor that can increase adaptability by protecting them from the psychological stresses that may appear in the adaptation process.

Additionally, life satisfaction has been shown to have a negative impact on the increase in depression over time. This is consistent with a two-year follow-up study of 189 NKRs [53], which showed that participants’ depression increased while their overall life satisfaction decreased. Another study of NKRYs also reported significantly lower life satisfaction in a group with depression than in a group without depression [19]. Several studies on refugees and immigrants mention post-immigration factors, such as experiencing discrimination, not having close friends, and acculturation stress, which affects life satisfaction or depression rather than pre-immigration stress [54,55,56]. Therefore, the results imply that depression prevention programs should consider ways to enhance resilience and life satisfaction and foster ego strength by recognizing emotions and promoting healthy emotional expression.

Meanwhile, external factors were not significant in the change in depression over three years in the present study. Specifically, psychological and practical support did not directly affect the change in depression. This is contrary to previous studies, which showed that the perception that support can be gained from relatively close family or peer relationships plays a positive psychological role in the cultural adaptation process [23,45,57]. However, according to a study by Jeong and Kang [8] on environmental protection factors, peer support alone does not have a significant impact on cultural adaptation stress, but can be indirectly influenced by personal internal protection factors such as internal locus of control, suggesting the indirect influence of external factors. Regarding family function, no statistically significant association with changes in depression over time was reported in this study. Meanwhile, Nam et al. [24] revealed that family cohesion was significantly associated with depression among NKRs, in contrast to our finding that family adaptability was not associated with depression. However, half (50%) of the participants were reported to be living with friends or in dormitories away from their families, and may therefore be significantly affected by peer relationships rather than family relationships [58].

There are some limitations to this study. First, the sample size was relatively small; therefore, care should be taken in interpreting and applying the results. Given the characteristics of school-based research, the dropout rate was very high because of changes in students enrolled in the school; participants who failed the study were no longer present at school due to reasons such as graduation or suspension of study, and were no longer able to follow up. This was beyond the control of the researchers; therefore, we propose in following studies the need to select a study subject by considering the dropout potential of participants in the research design phase. Second, some scales, such as psychological support and practical support, comprised a single question, which had limitations for obtaining substantial responses. Therefore, future studies will likely need to gather more detailed information through validated questionnaires. Also, the Cronbach’s alpha for the measure of expressive suppression was relatively low. We believe this is due to the small total sample size, and one must be careful about understanding the result. Third, although this study explored variables that affect temporal changes in depression, there are limitations that have not been able to compare the relative influence of variables or present structural models. Therefore, for further studies it is recommended to use statistical methods to clarify the temporal causal relationship.

Nevertheless, to the best of our knowledge, there is only one longitudinal study of NKRYs to date [16], and it is therefore a significant achievement for this study to identify individual psychological changes over three years using follow-up observations. It is also meaningful in that it provides information on the mental health of NKRYs in the initial settlement process, less than five years after settlement. In this study, we found a tendency for depression in NKRYs to increase year by year for three years. Emotional suppression has also been found to be a risk factor that can increase depression, and resilience and life satisfaction have been shown to be protective factors that decrease depression. These results suggest that the development of programs to encourage individual resilience and life satisfaction is required to promote mental health in NKRYs, and to seek ways to promote and safely address the emotions experienced. Further research is required to expand and justify the research results through a larger and more representative sample.

## Figures and Tables

**Table 1:** Characteristics of participants at the baseline point.

Characteristics	N = 64
Gender, N (%)	
Male	24 (37.5)
Female	40 (62.5)
Age (years), mean (SD)	16.89 (1.64)
Periods of living in South Korea (years), mean (SD)	3.06 (2.54)
Birth place, N (%)	
North Korea	22 (34.4)
China	42 (65.6)
Residential type, N (%)	
Living with their families or relatives	32 (50)
Living alone or with their friends or in facilities	32 (50)
Childhood trauma experience, mean (SD)	1.63 (2.38)

Note SD: standard deviation accounted for.

**Table 2:** Characteristics of variables, mean (SD).

Variables	1st Year	2nd Year	3rd Year	F
Depression	19.88 (9.07)	22.69 (11.18)	22.86 (10.92)	3.09 *
Cognitive reappraisal	19.58 (3.08)	19.89 (3.27)	20.70 (3.58)	2.59
Expressive suppression	12.14 (2.45)	12.16 (2.37)	12.13 (2.61)	0.004
Resilience	18.56 (4.65)	19.22 (4.1)	18.34 (4.84)	1.594
Life satisfaction	5.52 (2.15)	5.38 (2.13)	5.34 (2.26)	0.196
Psychological Support	6.69 (2.74)	6.56 (2.4)	6.58 (2.09)	0.076
Practical Support	7.44 (2.47)	6.66 (2.23)	6.75 (2.32)	3.516 *
Family adaptability	29.75 (7.19)	29.81 (6.76)	30.14 (6.8)	0.107
Family cohesion	32.63 (7.83)	32.28 (7.54)	32.11 (7.92)	0.209

* *p* < 0.05.

**Table 3:** The result of random-effects GLS regression.

Variables		Coef.	Std. Err	Z	*p* > lzl
Control Variables	Gender	1.647	1.894	0.87	−0.385
	Age	0.375	0.443	0.85	0.40
	Birth place	0.166	1.860	0.09	0.93
	living in South Korea	−0.235	0.301	−0.78	0.44
	Residence type	0.701	0.867	0.81	0.42
	ACE	0.196	0.363	0.54	0.59
Intra-personal Factors	Cognitive reappraisal	−0.240	0.200	−1.20	0.23
	Expressive suppression **	0.886	0.282	3.14	0.002
	Resilience ***	−0.867	0.176	−4.92	0.000
	Life satisfaction *	−0.798	0.361	−2.21	0.027
External Factors	Psychological Support	−0.059	0.331	−0.18	0.860
	Practical Support	−0.071	0.343	−0.21	0.837
	Family adaptability	−0.070	0.178	−0.39	0.697
	FamilyCohesion	0.026	0.174	0.15	0.882
_cons		28.06	11.07	2.54	0.01

Note: Coef: coefficient, Std. Err: standard error, * *p* < 0.05, ** *p* < 0.01, *** *p* < 0.001.

## Data Availability

The data presented in this study are available on request from the corresponding author. The data are not publicly available due to privacy.

## Supplementary Materials

The following are available online at https://www.mdpi.com/1660-4601/18/4/1696/s1, Table S1: Correlation analysis between depression and main variables.
