# Supplementary material for: Factors Affecting Changes in the Mental Health of North Korean Refugee Youths: A Three-Year Follow-Up Study
Source: Int J Environ Res Public Health. 2021 Feb 10;18(4):1696. doi: 10.3390/ijerph18041696 (PMC7916609; doi:10.3390/ijerph18041696)
Supplement: Supplementary file 1 [file ijerph-18-01696-s001.pdf]

Supplementary Table S1. Correlation analysis between depression and main variables

	T1 Expressive suppression	T2 Expressive suppression	T3 Expressive suppression	T1 Resilience	T2 Resilience	T3 Resilience	T1 Life satisfaction	T2 Life satisfaction	T3 Life satisfaction
T1 Depression	0.216 (0.086)	0.187 (0.140)	0.089 (0.483)	-0.576*** (0.000)	-0.457*** (0.000)	-0.401*** (0.000)	-0.194 (0.124)	-0.240 (0.057)	-0.046 (0.719)
T2 Depression	0.095 (0.455)	0.432*** (0.000)	0.091 (0.474)	-0.407** (0.001)	-0.546*** (0.000)	-0.437*** (0.000)	-0.361** (0.003)	-0.508*** (0.000)	-0.286* (0.022)
T3 Depression	-0.010 (0.938)	0.266* (0.034)	0.096 (0.451)	-0.311* (0.012)	-0.516*** (0.000)	-0.501*** (0.000)	-130 (0.306)	-0.272* (0.030)	-0.371** (0.003)

Pearson's *r* (*p*-value) was presented.

**p* < .05, ***p* < .01, ****p* < .001.
